# An Improved Time-Series Model Considering Rheological Parameters for Surface Deformation Monitoring of Soft Clay Subgrade †

**DOI:** 10.3390/s19143073

**Published:** 2019-07-11

**Authors:** Xuemin Xing, Lifu Chen, Zhihui Yuan, Zhenning Shi

**Affiliations:** 1 Laboratory of Radar Remote Sensing Applications, Changsha University of Science & Technology, Changsha 410014, China; 2 School of Traffic and Transportation Engineering, Changsha University of Science & Technology, Changsha 410014, China; 3 School of Electrical and Information Engineering, Changsha University of Science & Technology, Changsha 410014, China; 4 School of Engineering, Newcastle University, Newcastle upon Tyne NE1 7RU, UK

**Keywords:** deformation model, time series deformation, rheological parameter, highway

## Abstract

Building deformation models consistent with reality is a crucial step for time-series deformation monitoring. Most deformation models are empirical mathematical models, lacking consideration of the physical mechanisms of observed objects. In this study, we propose an improved time-series deformation model considering rheological parameters (viscosity and elasticity) based on the Kelvin model. The functional relationships between the rheological parameters and deformation along the Synthetic Aperture Radar ( SAR) line of sight are constructed, and a method for rheological parameter estimation is provided. To assess the feasibility and accuracy of the presented model, both simulated and real deformation data over a stretch of the Lungui highway (built on soft clay subgrade in Guangdong province, China) are investigated with TerraSAR-X satellite imagery. With the proposed deformation model, the unknown rheological parameters over all the high coherence points are obtained and the deformation time-series are generated. The high-pass (HP) deformation component and external leveling ground measurements are utilized to assess the modeling accuracy. The results show that the root mean square of the residual deformation is ±1.6 mm, whereas that of the ground leveling measurements is ±5.0 mm, indicating an improvement in the proposed model by 53%, and 34% compared to the pure linear velocity model. The results indicate the reliability of the presented model for the application of deformation monitoring of soft clay highways. The estimated rheological parameters can be provided as a reference index for the interpretation of long-term highway deformation and the stability control of subgrade construction engineering.

## 1. Introduction

Stability control of highways built on a soft clay subgrade is one of the key technical problems for highway subgrade engineering. Due to the geological characteristics of large natural moisture content, high compressibility, low strength, and poor structure of soft clay, roads built on soft clay subgrade are more prone to displacement and instability, especially under large traffic loads. Consequently, long-term surface deformation monitoring for infrastructure built on soft clay, after highway embankment settlement construction, is of considerable practical significance to the prevention of transportation safety accidents and the assurance of highway construction quality [1,2]. Although differential interferometric synthetic aperture radar (DInSAR) can cover a shortage of traditional ground measurement methods, its capacity in highway deformation monitoring is limited by its well-known spatial-temporal decorrelation and atmospheric delay effects [3,4]. Time-series technologies, such as permanent scatterer interformetry (PSI) [5], small baseline subset (SBAS) [6,7], temporally coherent point InSAR (TCP-InSAR) [8], and so on [9], have been proven to possess great capacities for large traffic infrastructure monitoring (i.e., railways, highways, and bridges) [10,11,12,13,14,15,16,17,18,19,20,21]. They can pick up ground displacement information with millimeter-level precision through high coherence points, maintaining long-term stable backscatter characteristics. Thus, they are insusceptible to spatial-temporal decorrelation [22].

Deformation modeling is a crucial step in time-series processing, determining the temporal and functional relationships between the phase component of displacement and the deformation parameters over highly coherent points. An accurate and reliable deformation model can not only improve the accuracy of deformation estimation, but also control the residual phase within a reasonable range of a whole phase cycle [−π, π]. Deformation modeling has a significant impact on the subsequent processing steps, including high coherence point identification, unknown parameter estimation, and phase unwrapping. It can also provide a reference for the interpretation of the final deformation results. Among traditional time-series models, the most commonly used is the linear velocity model, which simply assumes that temporal displacement follows linearly varying characteristics, and treats the deformation rate as a constant parameter over each time-adjacent interferometric period. This model was originally proposed as a PSI technique, and has been successfully applied in a large amount of cases. However, under the assumption of a pure linear varying characteristic among all temporal periods, the linear velocity model has significant limitations. When the real deformation of the monitored object is close to a linearly varying characteristic, the residual phase can be easily suppressed within the range of a whole phase cycle; however, when a strong non-linear component exists in the total displacement, the residual phase may possibly exceed the reasonable range of [−π, π], thus inducing a non-unique solution of unknown parameters and large inaccuracy. Due to the deficiency of the linear model, some non-linear deformation models have been presented, such as the Seasonal [23,24], Polynomial [25], Hyperbola, and Spline function [26] models. Although these models have achieved fitting of the temporally varying process of deformation for different observed features with better experimental results, they are generally based on a combination of one or several empirical mathematical functions to fit the deformational variations, ignoring the physical mechanism of deformation of the monitored object. The parameters for those models are generally mathematical coefficients that lack physical significance. Soft clay has the properties of mellow soil, large natural water content, and high compressibility. Under the conditions of constant external load, deformation is related to natural compression and the extravasation of the inner water in the soft soil, combined with external environmental factors (such as rainfall and temperature), thus the deformation of soft clay is characterized as an obvious temporal non-linear variation. In particular, for highways built on soft clay, a single pure empirical mathematical function may not describe the actual dynamic evolution, due to its temporally complicated non-linear characteristics, and a negative impact could be imposed on the accuracy of the obtained measurements and the subsequent displacement prediction. This would be adverse to the corresponding long-term analysis and deformation interpretation following highway construction.

According to authoritative statistics, more than 70% of pavement structure damage is related to long-term rheological deformation of the subgrade [27]. The rheological property is one of the primary engineering properties of soft soil, representing the temporal effect of soil deformation. Rheology is a subject that studies the deformation laws of materials over time under certain conditions (e.g., stress and strain) [28]. Rheological parameters (elastic modulus and viscosity) are significant factors for characterizing the rheological properties of soft clay. During the operation step of highway post-construction, the external load can be considered as constant and the underground deformation increases with time, so the rheological deformation plays a dominant impact role. In the theory of rheology, the rheological model is a kind of mechanical model (composed of spring, dashpot, and slide rod) that represents the rheological characteristics of rocks and soil and describes the dynamic temporal evolution process. The most widely used rheological models can be divided into linear models and non-linear models. For linear rheological models, a qualitative analysis of the material is initially carried out, then the corresponding rheological state function is constructed, which quantitatively represents the functional relationship between the strain of soft soil material and physical variables (i.e., viscosity, elastic modulus, and time). These are mainly based on the series-parallel connection of basic mechanical components (i.e., Burgers model, Kelvin model, and Maxwell model, among others). This kind of model can easily and intuitively express complex mechanical properties, which is helpful for conceptually understanding the elastic and visco-elastic properties of soft soil deformation. Their mathematical expressions can directly describe the rheological deformation, and are applicable to the simulation of the initial or stable rheological deformation of rock and soil material [29,30]. However, theoretical models for time-series displacement that consider rheological parameters have been rarely mentioned in previous InSAR deformation studies.

Based on the background discussed above, we propose a time-series deformation model based on rheological theory. The Kelvin rheological model, a typical one-dimensional linear rheological model, is adopted to form a functional relationship between radar line-of-sight deformation and the rheological parameters (elastic modulus and viscosity). The method of rheological parameter estimation is also illustrated in this paper. The proposed model is tested by a simulated experiment and a real data experiment. In the real data scenario, the rheological parameters of a stretch of highway (namely, the Lungui Highway in Foshan, China) are obtained, and the time-series subsidence over the period of June 2014 to December 2015 is investigated using TerraSAR X imagery.

## 2. Time-Series Modeling Considering Rheological Parameters

### 2.1. Time-Series Deformation Model

Suppose M+1 SAR images covering the same area are acquired in repeat orbits at different dates. Then, N interferometric pairs may be generated, according to certain temporal baseline and spatial baseline thresholds, where the N interferometric pairs are generated through two-orbit D-InSAR processing, while satisfying the inequality $\frac{M}{2}\leq N\leq \frac{M\left(M-1\right)}{2}.$ In the processing, all images are registered and resampled to the same image first. Then, an external DEM is used to remove the topographic phase and, consequently, phase unwrapping is carried out for each interferometric pair. For each high coherence point P in the *i*-th interferogram, the wrapped interferometric phase can be expressed as [31]:

(1) $$\Delta \varphi_{i}^{p}=\frac{4\pi }{\lambda }\Delta d_{i}+\Delta \varphi_{i}^{topo,p}+\Delta \varphi_{i}^{res,p}$$

where λ is the radar wavelength (the X band is used in our real data experiment, and the corresponding λ is 3.2 mm); Δd is the line-of-sight cumulative deformation over the time period of the *i*-th interferogram, indicating the low-pass (LP) component of the total deformation; $\Delta \varphi_{i}^{topo,p}$ represents the residual topographic phase component, which can be expressed a $\Delta \varphi_{i}^{topo,p}=\frac{4\pi B_{i}}{{\lambda R}_{p}\sin\theta }\Delta Z^{p}$, $\mathrm{where}\;B_{i}$ defines the vertical baseline, θ is the incident angle, $R_{p}\;\mathrm{represents}\;\mathrm{the}$ distance between the sensor and the target $P,\;\mathrm{and}\;\Delta Z^{p}$ defines the residual elevation, which is an unknown parameter; and $\Delta \varphi_{i}^{res,p}$ is the residual phase component, including phase noise, atmospheric delay, and the high-pass (HP) deformation component. Taking the pure linear model as an example, $\Delta d_{i}$ in Equation (1) can be written as

(2) $$\Delta d_{i}=\;v\;t,$$

where v is the linear deformation rate, which is regarded as a constant parameter over each time-adjacent interferometric period, and t defines the temporal baseline for the *i*-th interferogram.

### 2.2. Rheological Model Based on the Kelvin Model

**Figure 1:**
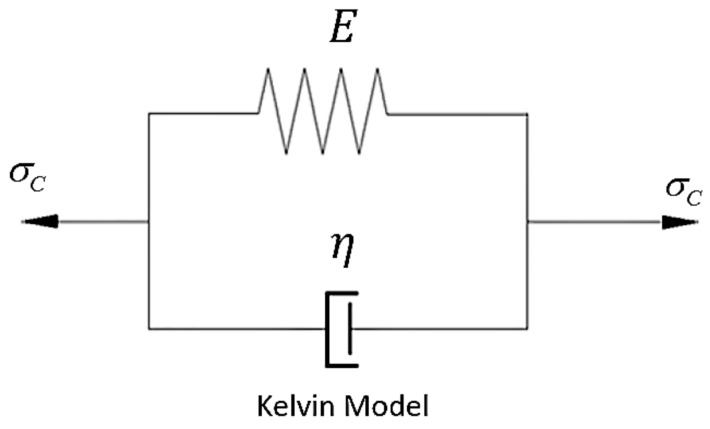
Kelvin rheological model (with a constant external load $\sigma_{c}$).

As discussed above, a Linear rheological model can express the complex mechanical properties of the rheological deformation easily and directly. Consequently, a one-dimensional linear rheological model, the Kelvin model, was selected for our experiments. The Kelvin rheological model is a kind of commonly used delay model, based on mechanical composition elements. It is a parallel system with a spring (pure elastomer) and a glue pot (pure viscous body), illustrating the phenomenon that, under the action of stress, the strain of the material does not reach the final strain value immediately, but has a relative lag process. Figure 1 shows a schematic diagram of the combined elements in the Kelvin rheological model. The rheological state equation of the Kelvin model can be written as [32]:

(3) $$\varepsilon =\frac{\sigma_{c}}{E}\left(1-e^{-\frac{E}{\eta }t}\right)$$

where ε defines the strain related to the material and $\sigma_{c}$ defines a constant external load. When the post-construction operation stage of a highway starts, the external load mainly includes the gravity of the surface layer and the load of the traffic vehicles. However, for practical analysis, the load of the vehicles can be ignored, due to its minor magnitude relative to the gravity of the highway layer. The gravity of the highway layer can be obtained through the investigation of the highway structure and soil mass sample testing in the upper part of the soft soil layer. E represents the elastic modulus of the material, which is also called the deformation modulus; whereas η defines viscosity, also known as the viscosity coefficient. E and η are significant rheological parameters, which are treated as unknown parameters in Equation (3). Finally, *t* represents the total time span of strain occurrence.

The functional relationship between the subsidence of the soft clay subgrade $S_{v}$ and the strain ε can be expressed as [28]:

(4) $$S_{v}=\overset{t_{2}}{\underset{t_{1}}{\int }}\overset{H}{\underset{0}{\int }}\varepsilon \cdot dhdt,$$

where *H* is the average thickness of the soft soil layer, which can be obtained by consulting the highway design materials; and h and *t* are integral variables, representing the soft clay thickness and the time span of subsidence, respectively. The subsidence ${\;S}_{v}$ can be further written as

(5) $$S_{v}=\frac{H\sigma_{c}}{E}\left(t_{2}-t_{1}\right)-\frac{\eta H\sigma_{c}}{E^{2}}\left(e^{-\frac{E}{\eta }t_{1}}-e^{-\frac{E}{\eta }t_{2}}\right)$$

### 2.3. Improved Deformation Model Considering Rheological Parameters

When horizontal movement is ignored, the vertical settlement can be calculated according to the formula $S_{\mathrm{LOS}}{\;=\;S}_{v}\cos\theta$. Combined with Equations (3)–(5), the deformation components related to rheology along the line-of-sight direction can be expressed as

(6) $$S_{LOS\_rhe}=\frac{H\cos\theta \sigma_{c}}{E}\left(t_{2}-t_{1}\right)-\frac{\eta \cos\theta H\sigma_{c}}{E^{2}}\left(e^{-\frac{E}{\eta }t_{1}}-e^{-\frac{E}{\eta }t_{2}}\right).$$

For each interferogram, $t_{1}$ and $t_{2}$ represent the acquisition date of master and slave images, respectively. Equation (6) is introduced into the original time-series deformation model, and the low-pass deformation component of the model can be rewritten as a combination of linear and rheological components:

(7) $$S_{LOS}=v\left(t_{2}-t_{1}\right)+S_{LOS\_rhe}$$

After substituting Equation (7) into Equation (1), the phase in Equation (1) can be expressed as

(8) $$\Delta \varphi_{i}^{p}=\frac{4\pi \cos\theta }{\lambda }\left[\frac{H\sigma_{c}}{E}\left(t_{2}-t_{1}\right)-\frac{\eta H\sigma_{c}}{E^{2}}\left(e^{-\frac{E}{\eta }t_{1}}-e^{-\frac{E}{\eta }t_{2}}\right)\right]+v\left(t_{2}-t_{1}\right)]+\frac{4\pi B_{i}}{{\lambda R}_{p}\sin\theta }\Delta Z^{p}+\Delta \varphi_{i}^{res,p}\;.$$

Suppose there are N interferometric pairs generated, and that the unknown parameters in the Equation are the rheological parameters E and η, linear rate  v , and elevation correction ΔZ. Supposing that there are at least four interferometric pairs generated, the unknown parameters over all high coherent points of each image can be solved and, consequently, the corresponding rheological parameters can be estimated.

### 2.4. Unknown Parameter Estimation

The estimation of the unknown parameters in Equation (8) is a non-linear parameter estimation problem. The genetic algorithm for non-linear least-squares estimation is utilized here to estimate the unknown parameters. The genetic algorithm is a method based on global optimization searching that is insusceptible to both the number of unknown parameters and the specific form of the model. The basic idea of the genetic algorithm is to obtain the population individuals as the final solution of the parameters, which can satisfy the condition of minimizing the fitness function through the operations of selection, crossover, and mutation. The population size, iteration times, and individual gene magnitudes for each individual of the population need to be set preliminarily [33]. According to Equation (8), each individual gene of a population includes the rheological parameters (E and η), linear velocity v, and elevation correction ΔZ. The fitness function is mainly modeled following the residual minimum norm principle, which can be expressed as follows:

(9) $$f\;=||\Delta \varphi_{i}^{res,p}\left|\right|\;=\;\min,$$

where $\Delta \varphi_{i}^{res,p}$ represents the residual phase in Equation (8). The general search procedure includes the following steps. (1) The magnitude of each initial individual gene should be set, which means the initial value range of each parameter should be fixed, and the corresponding fitness function value of each individual population can be calculated. (2) Whether the iteration termination condition for the minimum fitness function is satisfied should be determined. If not, multiple steps of selection, crossover, and mutation should be carried out to generate a new population of individuals, after which the fitness function value will be calculated again. If it is satisfied, the generated individual genes will be selected as the final estimated parameter. (3) As mentioned in [34], the simplex method can improve the precision of the results generated by the genetic algorithm; thus, we introduce it into our experiment to optimize the searching results. The parameters obtained by the genetic algorithm are taken as the input initial value of the simplex method, and the output optimized searching results are determined as the final solutions.

## 3. Simulated Experiment

In order to verify the feasibility and accuracy for solving the aforementioned models, a simulated experiment was designed and implemented. The elastic modulus coefficient was set up by investigating the design materials and the structural morphology of the test highway. It was controlled within the interval  [0,50] MPa. The v iscosity η was set within the interval $\left[0,\;8\right]\times 10^{6}$ Mpa. The linear deformation velocity *v* was within the range of [−0.2,0.1] m/y. The real parameter fields of elastic modulus, viscosity, and linear velocity were simulated by a two-dimensional Gaussian function model. The elevation correction ΔZ was simulated through a Gaussian random simulator, with the value controlled within the range of [−50, 50] m. Linear velocity was simulated using the Matlab peaks function, which can satisfy both positive and negative distribution characteristics of displacement [35]. There were 200 high coherence points and 10 interferograms generated in the simulation. With the known SAR sensor parameters (TerraSAR-X Stripmap data with descent orbital mode was used), including spatial and temporal baselines of each interferometric pair, values of all the parameters for over 200 high coherence pixels could be detected from the simulated field as true values in the following validation.

**Figure 2:**
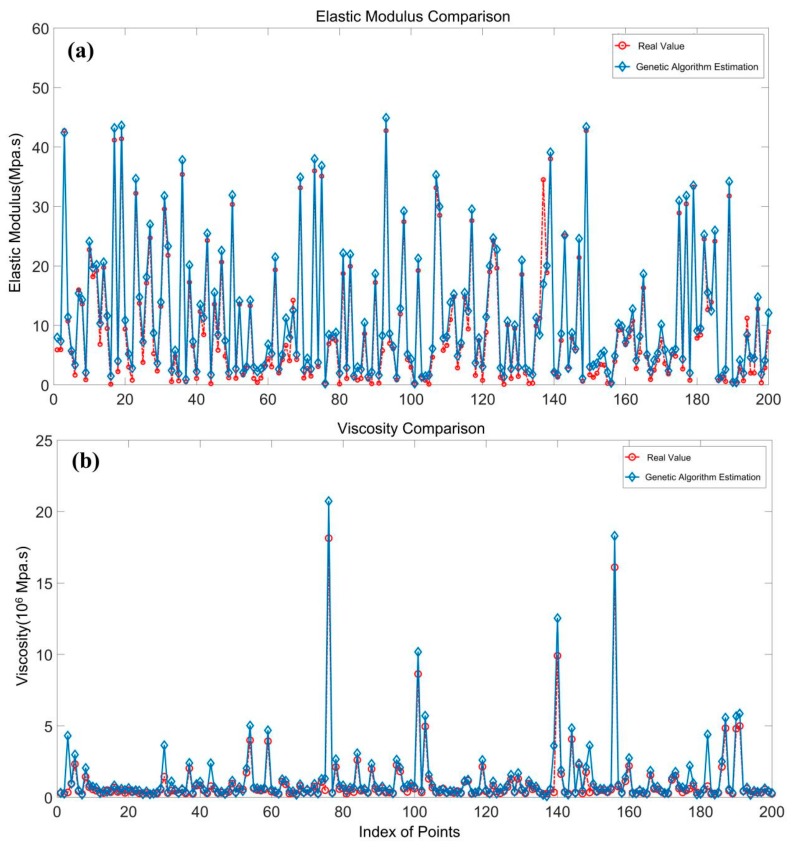
Estimated rheological parameters compared with real values in the simulation (the noise level is 0.5 rad).

With the initial estimation of the unknown parameters obtained by the genetic algorithm, the simplex method was used to determine the final solutions. Compared with the real values detected from the simulated field, the accuracy of the model and algorithm were evaluated. Figure 2 shows the comparison between the estimated value of rheological parameters and the real values (the noise level here was 0.5 rad). From Figure 2, we can see that the red and blue broken lines show good consistency, indicating that the estimated parameters were in good agreement with the true values. Table 1 shows the quantitative comparisons of RMSE (root mean square error) for each unknown parameter in Figure 2. For the four unknown parameters, the magnitude of errors accounted for lower than 6% of the mean parameter estimations. The comparative results imply the feasibility and reliability of the aforementioned model and parameter estimation method.

## 4. Real Data Analysis

### 4.1. Geological Background of Study Area

**Figure 3:**
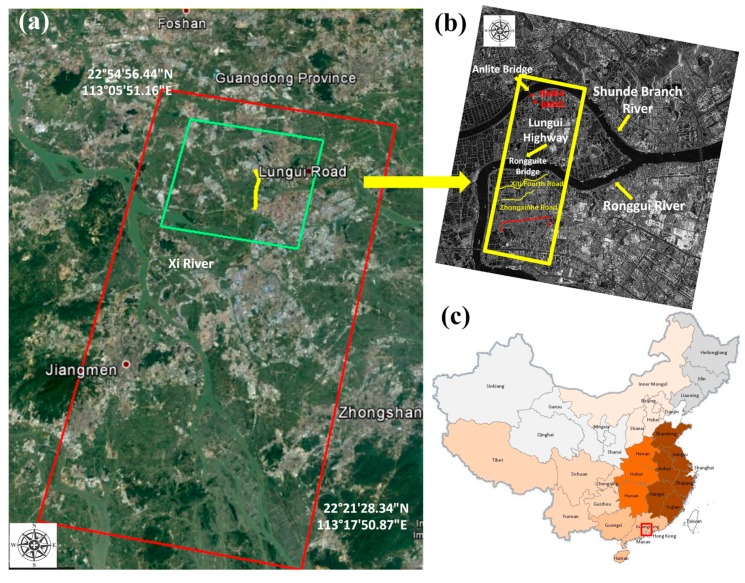
(**a**) Regional scale in China; (**b**) an amplified image of the area within the highway region of interest (outlined in the yellow rectangle); and (**c**) location of the study area in China.

The test area selected in this paper was a stretch of a highway; namely, the Lungui Highway, located in Shunde district, Foshan city, Guangdong province, China. The construction of the Lungui road started in March 2011. It was opened to traffic segmentally during the construction process, and the whole route officially opened to traffic in January 2015. The Lungui Highway connects Longzhou Road, Nanguo Road, and Hengjiu Road northward, becoming one of the most important connection channels between the Shunde west district and the three main routes (from south to north) in Shunde Central City. Figure 3a shows the study area, featured at different scales. As Figure 3a shows, the red rectangle outlines the spatial coverage of the selected TerraSAR-X images, while the green rectangle shows the subset for generating the interferometric results. Figure 3b shows the location of the Lungui Highway with the average intensity map as background. As shown in Figure 3b, the Lungui Highway is located close to three hydrological systems: the Xi River and the Rongui and Shunde Branch Rivers. Plenty of ponds and a large amount of silt are distributed along the route.

According to the design criteria of the test highway, the permissible vertical post-construction settlement is 30 cm/y for regular road segments, 20 cm/y for culverts, and 10 cm/y for bridge connections. According to the statistics of the Fuoshan Transportation Bureau, the passenger flow volume in 2014 of the Shunde District, where the highways are located, was up to 2018.31 million people per kilometer, whereas the freight flow on the test highways was approximately 654.64 million tons per kilometer. This huge traffic flow indicates the significant traffic situation of the Lungui Highway. According to our collected geological material, with a developed surface water system and extensive aquifers, the soft soil covering the upper layer is extremely soft and has high compressibility. Delta alluvial and silt plain dominates the topography of the area. Due to these geological characteristics, the subgrade of the highway is extremely prone to liquefaction and seismic subsidence. For this reason, long-term stability monitoring of this area is critically necessary. The yellow rectangle in Figure 3b defines the test stretch of Lungui road of interest in our experiment. Two major bridges, namely the Rongguite and Anlite Bridges, are contained in the test highway. Figure 3c shows the corresponding location of the test area on the China map.

**Figure 4:**
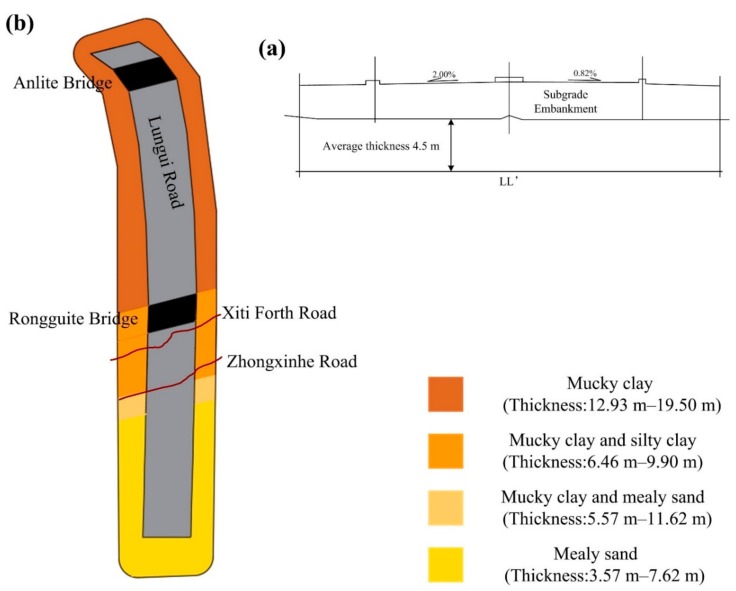
(**a**) Transversal profile at LL’ in Figure 3a and (**b**) geological distribution of the soft soil along the longitudinal direction of the Lungui Highway.

Figure 4 shows the transversal profile of section LL’ in the test area (see the red solid line at the bottom of Figure 3b), where 2% and 0.82% represent the gradient, and the average thickness of the soft soil layer in this cross-section is 4.5 m. From the transversal distribution along the test highway, the main distribution characteristics of the geotechnical layer are as follows: ground layer distribution is pseudo-viscosity plain fill and a quaternary system of brand-new sea-land cross stratum. The surface quaternary is mainly composed of silty soil and mealy sand, deposited by sea and land, including mucky clay, silty soil, and mealy sand. The underground strata below the Rongguite and Anlite Bridges are mainly argillaceous siltstone and silty mudstone. From the longitudinal distribution characteristics of the route, the soft soil layer in the north of Rongguite Bridge is mainly composed of continuously distributed mucky clay, with a thickness of 12.93–19.50 m. In the section between Ronguite Bridge and Xiti Fouth Road, the main components of the soft soil are mucky clay and silty clay, with a thickness of 6.46–9.90 m. In the section between Xiti Fouth Road and Zhongxinhe Road, mucky clay and mealy sand dominate the soft clay layer, with a thickness of 5.57–11.62 m. In the last section, south of Zhongxinhe Road, the soft layer is mainly mealy sand, with a thickness of 3.57–7.62 m (as shown in Figure 4b).

### 4.2. SAR Acquisition and Data Processing

**Figure 5:**
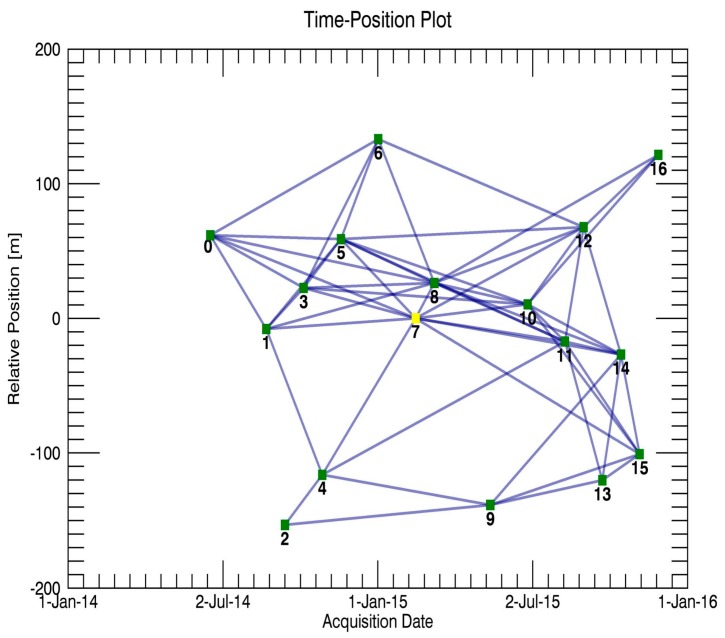
Temporal and perpendicular baselines of the available pairs.

**Figure 6:**
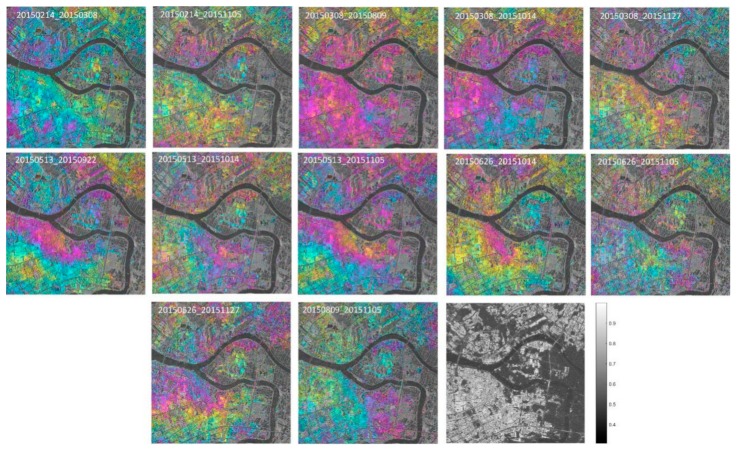
Selected interferometric images and the average coherence map (the last picture, bottom right) of the area shown in Figure 3a.

A total of 17 repeat-pass TerraSAR X-band Stripmap images were collected (orbit no. 119, descending), with a spatial resolution of 3 m (3.29 m along azimuth, 2.64 m along range, average incidence angle of 26.4°). These acquisitions covered the period from 17 June 2014 to 27 November 2015. The parameters of these TerraSAR-X images are listed in Table 2. In the processing of the two-pass differential interferometry, a subset of 18 × 15 km was selected, covering about a quarter of the total area (see Figure 3a). SBAS processing was used to generate the unwrapped interferograms for the test area. Due to the narrow ribbon characteristics of our observed object, the multi-look ratio along range and azimuth directions was set as 1:1 to ensure the original resolution of the test highway. The thresholds for the temporal-spatial baseline of the interferometric combination were empirically set to 130 m and 300 days, respectively. SARScape 5.2 and Envi 5.3 were used in our experiment to generate a total of 57 small baseline interferometric pairs. Figure 5 shows the spatial and temporal baseline for all the interferometric combinations in our experiment. The numbers 0–16 in Figure 5 correspond to each SAR image, and number 7 represents the index of the selected super master image (acquired on 14 February 2015). In the two-pass D-InSAR processing, all the rest of the images were registered and resampled to the super master image. In order to remove the topographic phase, a 1-arc-second Shuttle Radar Topography Mission digital elevation model (SRTM DEM, ~30 m spacing) provided by NASA was utilized. In addition, a Gaussian filter was selected to suppress the phase noise. After the flat earth phase removal and phase filtering processing, a polynomial fitting method was used to remove the orbital error and, then, the commonly used minimum cost flow (MCF) method was utilized to unwrap the wrapped interferometric deformation phases [36]. Finally, a total of 57 unwrapped interferometric images were generated. Figure 6 shows the selected interferometric images and the average coherence map.

**Figure 7:**
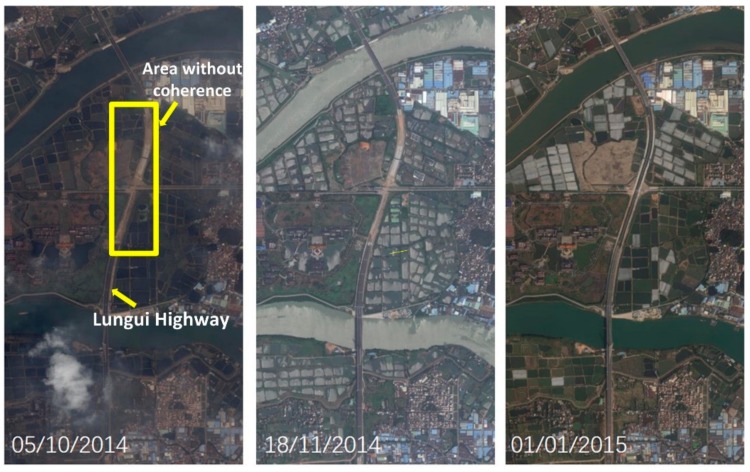
Study area on multi-temporal google maps. The area within the yellow rectangle was still under construction until December 2015.

During processing, high coherence candidates were selected, based on a coherence threshold of 0.6. In order to ensure that the most coherent points were distributed over the highway among the 57 total interferometric pairs, we selected the interferograms carefully and deleted those with bad coherence and less points along the route. Consequently, only 25 high-quality pairs with densely distributed coherence pixels over the highway region were selected. The subsequent experiments, including rheological parameter estimation and time-series deformation inversion, were carried out using MATLAB. Due to the large amount of densely distributed coherent points, the search operation of the genetic algorithm was extremely time consuming; thus, we masked the targets distributed along the route as our observed pixels. According to our in situ investigation and the design materials collected from the highway construction company, Fuoshan, China, we found a section of the test highway which was still under road surfacing from November 2014 to December 2014, and the whole route was opened to traffic in January 2015. We also downloaded the corresponding Google Earth maps covering the test area, which are shown as Figure 7. As shown in Figure 7, from November 2014 to December 2014 the area located in the yellow rectangle was without a road surface, whereas, in the map acquired on January 2015, the surfacing was finished; thus, the highway was opened to traffic entirely at that time. In order to ensure the accuracy of our deformation results, we deleted the coherent points located in the yellow rectangle (due to their low coherence, the number of coherent candidates in the highlighted area was actually significantly lower than those in the other stretches of the highway). Finally, 6657 highly coherent points were selected.

The phase model of Equation (8) was established for each high coherence point, and the unknown parameters (v, E,η, and ΔZ) were obtained by the methods discussed in Section 2.4. Based on the investigation of geological data and rock structure characteristics in the test area, the initial individual gene range was set as follows: the elastic modulus coefficient E was set within the range of [0, 50] MPa, the viscosity η within the range of $\left[0,8\right]\times 10^{6}$ Mpa·s, the linear velocity v was in the interval [−0.5,0.2] m, and the elevation correction ΔZ was in the interval [−50,50] m. In the process of the genetic algorithm search, the upper threshold for the genetic population was set to 700 generations, with 1000 individuals in each population and a crossover probability of 0.7. The crossover mode was two-point crossover, and a Gaussian function was selected as the mutation function. The termination condition for the program iterations was minimization of the average fitness function value, which indicated a stable fitness value. Finally, the individual genes of population satisfying the fitness function value condition were selected as the estimated unknown parameters for the coherent point. Substituting the obtained final solutions of the unknowns into Equation (5), the low-pass (LP) component of time-series deformation (LP-deformation) could be acquired. Subsequently, in order to obtain the high-pass (HP) deformation component (HP-deformation), the residual phase in Equation (8) was processed with temporal high-pass filtering and spatial low-pass filtering [31]. The final time-series of the deformation were obtained through the sum of HP- and LP-deformation components on each pixel.

### 4.3. Experimental Results

**Figure 8:**
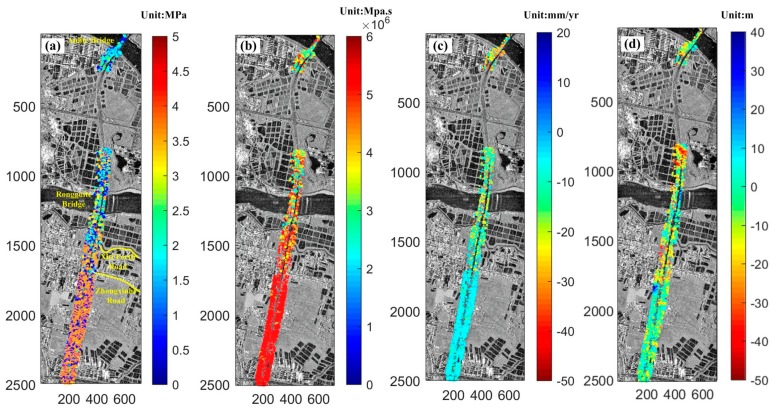
Estimated model parameters: (**a**) elasticity, E; (**b**)  viscocity, η; (**c**): linear velocity, v; and (**d**) height correction, ΔZ.

Figure 8 shows the results of the four unknowns, for all coherent points, in Equation (8). All images were in the slant-range projection. It can be seen, from Figure 8a,b that the elastic modulus was generally distributed within the range of [1.5, 5] Mpa, whereas the viscosity was distributed in $\left[2,\;6\right]\times 10^{6}\;\mathrm{Mpa}$. From the color distribution, both rheological parameters gradually varied near Xiti Fourth Road, whereas a clear color change boundary appeared next to Zhongxinhe Road. According to the field investigation of the area, from Xiti Fourth Road to Anlite Bridge along the route, breeding ponds (see the little black rectangles in Figure 8) and villages were densely distributed, and few typical urban buildings could be found near this stretch. The soil along this segment was mainly mucky clay and silty clay, as mentioned in Section 4.1. In contrast, the area below Zhongxinhe Road was generally urban districts, with densely distributed residential constructions including banks, office buildings, and other urban infrastructure. Correspondingly, the mucky content in the soil along this stretch was relatively low. Figure 8c shows the linear velocities in Equation (8), with an overall distribution of −50 to 20 mm/y. Similar color distribution characteristics can be found in the figure, with Zhongxinhe Road as an obvious boundary. The detected subsidence rate in the upper region was relatively obvious, with maximum value of 67 mm/y, whereas the area below Zhongxinhe Road was more stable, with the deformation velocity generally lower than 10 mm/y. Figure 8d shows the overall distribution of height corrections, and the results are generally within the interval of [−50,40] m, with a maximum DEM error of 95 m.

**Figure 9:**
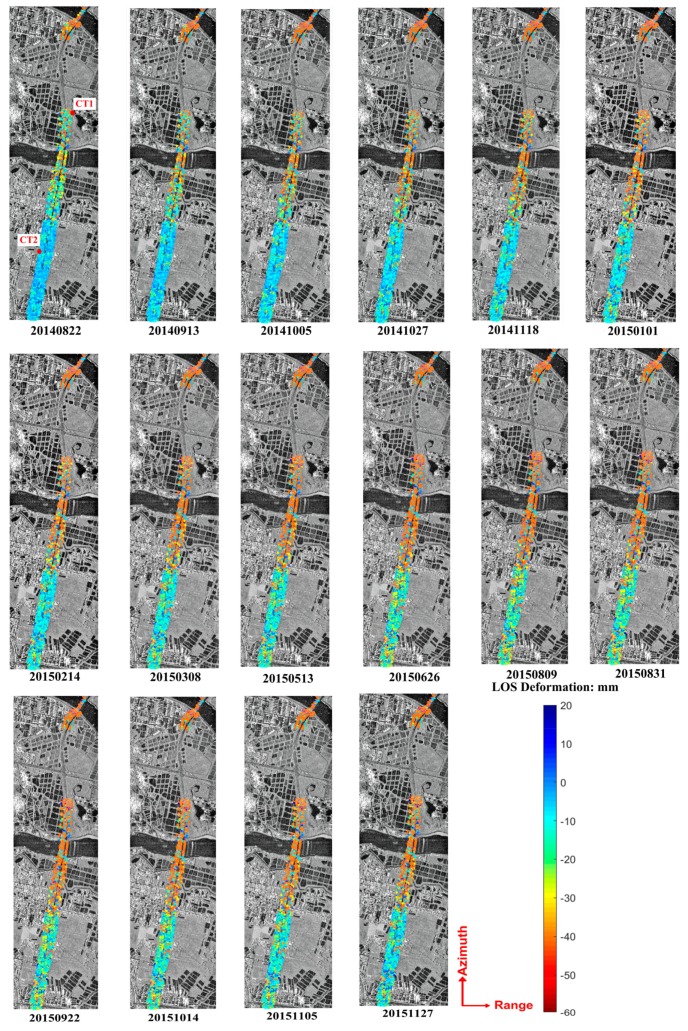
Time-series deformation over the tested area (with reference to 17 June 2014).

Figure 9 shows the overall time-series deformation results obtained for the test highway. From the spatial characteristics of the color distribution, we can see that Zhongxinhe Road is still the obvious dividing line in the images (with a dark orange color in the upper area), and that the maximum subsidence was 124 mm (on 27 November 2015). The deformation was significantly weaker in the bottom part (generally blue-green), with a maximum subsidence of only 48 mm. It can also be seen, from the temporal color variation in Figure 9, that the deformation was rapid subsidence from the initial time to May 2015, with the subsidence velocity decreasing slowly. However, from June 2015, the deformation showed a relatively stable performance, and even a slight uplift.

## 5. Discussions

### 5.1. Potential Reasons for the Deformation

According to the above analysis, the spatial characteristics of subsidence were related to the following:

(1). The magnitude of the obtained elastic modulus and viscosity parameters reflect the aforementioned deformation characteristics. It can be obviously seen, from Figure 8 and Figure 9, that the bottom area was under a relatively stable deformation, with higher elastic modulus and viscosity values. Under the condition of unidirectional stress, the elastic modulus equals the stress divided by the strain along the direction [37]. As Equation (4) shows, deformation can be understood as a temporal integration of strain. Therefore, when the external load is constant, the stress can be considered a constant, and the higher the elastic modulus is, the lower the deformation performs. The physical parameter viscosity (also known as the viscosity coefficient) is a measure to describe the viscosity of a fluid, which is a demonstration of the fluid flow dynamics for its internal friction phenomenon [38]. Higher viscosity reflects greater friction in fluid. In this paper, viscosity is treated as the parameter that reflects the internal friction property of soil mass and its ability to resist deformation. The higher the value of the viscosity, the greater the friction resistance between the soil mass is and, thus, less strain and deformation. This is the key reason why the areas with low deformation showed a higher magnitude of elastic modulus and viscosity.
(2). As described in Section 4.1, with densely distributed ponds around the upper stretch of the highway, mucky clay and silt dominated the geological content of the clay, and the soft soil layer of the upper segment was relatively thicker (with a thickness of 12.93–19.50 m). In addition, the water system around this stretch was well-developed and, thus, the ground subsidence was more obvious. In contrast, the areas below Zhongxinhe Road in the image were mainly urban districts, where the silt content in the soil of the road foundation was lower, with mealy sand and silty clay as the dominant geotechnical content. Furthermore, compared to the upper stretch of the highway, the average thickness of the soft soil layer was only 3.57–7.62 m, with a lower water discharge flow under the surface and an advanced drainage system in the urban areas. As a result, the settlement was much weaker.

### 5.2. Temporal Deformation Characteristics over Feature Points

**Figure 10:**
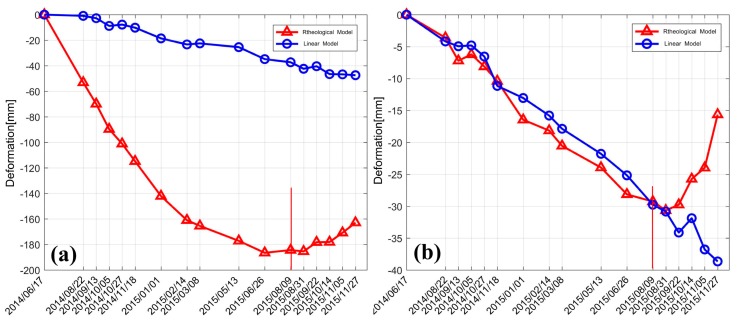
Time-series results on two feature points: (**a**) CT1 and (**b**) CT2 (with reference to 2014/6/17).

In order to further investigate the temporal variation characteristics of deformation, two feature points (CT1 and CT2) were selected for analysis (the locations are shown in the first image of Figure 9), with comparison to the results obtained through the pure linear model (see Figure 10). CT1 was located in the bottom area of the image, where the subsidence was quite obvious (with an accumulated subsidence up to 185 mm), whereas the maximum subsidence in the linear velocity model was only 47 mm. The deformation difference between the two models was mainly due to the large rheological component of the deformation obtained at point CT1. According to the estimation of Equation (8), the linear component at CT1 only accounted for 24% of the total deformation; in contrast, the rheological component accounted for 71%, and the residual deformation isolated from the residual phase accounted for 5%. As shown in the results of the pure linear velocity model, the linear deformation component accounted for 84% of the total deformation, and the non-linear part of the residual phase accounted for 16%. This indicates that a majority of the non-linear deformation may not be reflected in the residual phase of the linear velocity model when a substantial real non-linear deformation has occurred. Therefore, the obtained non-linear deformation result, isolated from the residual phase of the linear model, may have a large deviation from the real value; thus, it shows a significant difference from the rheological model. As shown in Figure 10, the overall deformation sequences obtained by the rheological model displayed an obvious non-linear trend, whereas temporally continuous linear subsidence characteristics were displayed by the linear model. As shown in Figure 9, CT2 was located in the bottom, relatively stable area, and the corresponding time-series deformation obtained through both models are shown in Figure 10b. Due to the relatively low residual phase component in the linear model and the low rheological deformation component estimated in the rheological model, the results for the two models at CT2 were consistent during the period of June 2014 to August 2015, with a maximum difference of only 7 mm, and a maximum subsidence of 32 mm for the rheological model.

It can also be seen, from Figure 10b, that the rheological model results showed a slow subsidence recovery from August 2015, with a magnitude of 14 mm at CT1 and 15 mm at CT2. The reasons for this are proposed to be related to the following:

(1). Soft clay has the property of mellow soil, large natural water content, and high compressibility. During the period of June 2014 to June 2015, under the conditions of constant external load, the void between the soil mass was being compressed and the inner water was being released; thus, the deformation during this period was characterized as obvious subsidence with a decreasing velocity.
(2). Second, as discussed in Section 4.2, a stretch in the middle of the test highway was still undergoing road surfacing from June 2014 to November 2014, and compaction of the soft soil layer in the middle section may have accelerated the subsidence phenomena in nearby stretches.
(3). With the passage of time, when the natural compression of the soil reaches its limit and the porosity ratio drops to the minimum, the deformation caused by the early external load and extravasation of the inner water in the soft soil ceases. Consequently, the subsequent deformation was mainly affected by external environmental factors. According to precipitation data provided by the Fuoshan Meteorological Bureau, the annual precipitation in 2015 was 2055.2 mm, 20% higher than previous years. The spatial and temporal distributions of annual precipitation were extremely asymmetric, being three or four times higher in October and December. Extreme weather events, such as thunderstorms, wind, hail, and tornadoes occurred frequently, and disasters induced by rainstorms and typhoons were obvious. Under the combined influence of high trough and low vortex, continuous precipitation had occurred in the city from 27 August 2015. The average rainfall amount was 127.3 mm in Fuoshan city, with 100–250 mm recorded at 65% of the automatic stations, and 250 mm at Shunde automatic station [39]. Due to the increase of rainfall, both water content and discharge in the water system correspondingly increased. With accelerated flow speed, perched ground water in the upper layer of the subgrade increased due to the impact of rainfall and the supply from the surrounding water system. This is the key reason we suppose as the cause of the subsidence recovery that occurred from August 2015.

### 5.3. Comparative Analysis with other Non-Linear Time-Series Models

**Figure 11:**
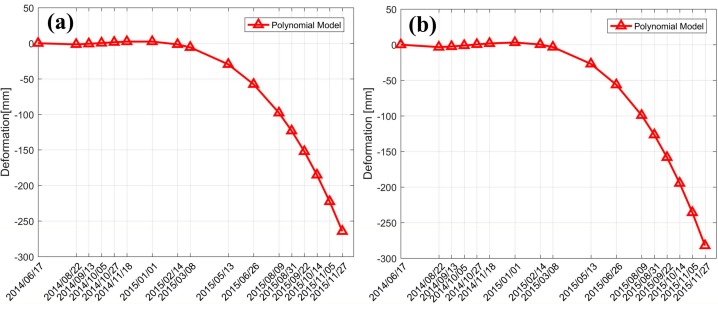
Low pass (LP)-deformation of the two feature points, derived from a polynomial model: (**a**) CT1 and (**b**) CT2 (with reference to 17 June 2014).

We also conducted an experiment based on a polynomial model to generate the time-series deformation over this route, according to [25]. The temporal displacement over the two feature points are shown in Figure 11 (we only showed the LP-deformation component). As Figure 11 shows, we can see that in the early stage (the period from June 2014 to February 2015) the temporal variation characteristic was a stable deformation velocity, whereas an obvious significant increase in deformation velocity was present in the later stage (the period from March 2015 to November 2015). As discussed above, the subsidence velocity should more reasonably follow a temporally gradual decrease for soft clay, which indicates that the polynomial model is not suitable here. Additionally, the accumulated displacements over both points were close to 300 mm over the test period, which exceeds the critical permissible maximum subsidence for a highway area (according to the design materials for the test road, the permissible subsidence is 20 cm). Our suggested reason for this incorrect result is related to the polynomial model itself. A polynomial model is a certain mathematical empirical modeling function with the significant advantage of spatial approximation. However, when the real temporal displacement variation does not follow the characteristics of a polynomial function, the estimated deformation results may be incorrect. Similar unexpected results may occur in other, similar time-series models (e.g., the logistic and hyperbolic models).

### 5.4. Accuracy Evaluation

**Figure 12:**
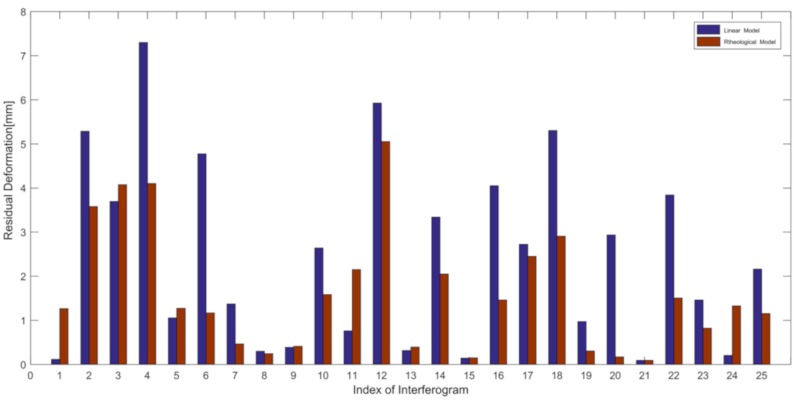
RMS of residual deformation of a 25-interferogram comparison for two models.

According to [25], the fitting accuracy of a deformation model can be reflected by the HP-deformation component. The smaller the HP-deformation is, the higher the accuracy of the selected model. The HP-deformation of each interferogram obtained through the rheological model was compared with that of the linear velocity model. Figure 12 shows a comparison of the average residual deformation over all high coherence points for each interferogram. It can be seen from the figure that all deformation was within 8 mm, where the deformation in the 2nd, 4th, 12th, and 18th interferograms was obviously higher, indicating that the residual phases of these images were large. For those images, the HP-deformation of the rheological model was obviously smaller than that of the linear model. For the rheological model, the variance of HP-deformation over all the interferograms was 1.6 mm, whereas that of the linear model was 2.4 mm, indicating an accuracy increase of 33% in the rheological model.

**Figure 13:**
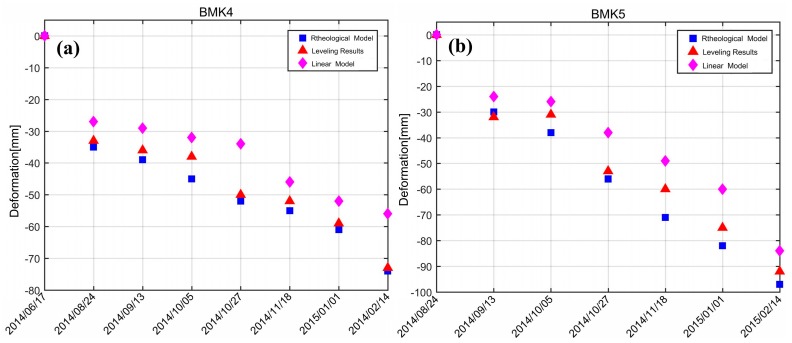
Time-series deformation results compared with leveling measurements on benchmarks: (**a**) BMK 4 in Figure 3a, and (**b**) BMK 5.

In addition, ground measurements of two leveling points in the test area were collected (the locations of the leveling points are shown in Figure 3a, close to Anlite Bridge). The temporal span of leveling measurement was from June 2014 to February 2015. In order to carry out an accurate comparison, we transferred the generated Line of Sight (LOS) deformation into vertical displacement according to the equation $S_{\mathrm{LOS}}{\;=\;S}_{v}\cos\theta$, and extracted the eight dates of the measurement data that coincided temporally with our SAR acquisition dates. The total reference point of leveling and all the SBAS processing methods were the same pixel, which was selected according to our in situ investigation and registered deformation material collection. The comparison results are shown in Figure 13, where the blue solid squares represent deformation results obtained by the rheological model, and the purple solid triangles illustrate the linear model results. It can be clearly seen in Figure 13 that the rheological model results were closer to the leveling results. Table 3 shows the quantitative comparison results of the root mean square error (RMSE) on the benchmarks. According to our calculation, the RMSE of linear model was ±10.7 mm, while the rheological model was ±5.0 mm, with an improvement of about 53%.

## 6. Conclusions

In this paper, a time-series deformation model considering rheological parameters was proposed, and the rheological parameters of elastic modulus and viscosity were introduced into a traditional empirical functional model. Based on the functional relationship between strain and time in the Kelvin rheological model, the function between LOS deformation and rheological parameters was established and the original linear deformation model was improved. The genetic algorithm method was used to solve for the initial values of the model parameters, and the simplex method was used for subsequent optimization. In order to verify the feasibility and reliability of the model and the parameter estimation algorithm, a simulated experiment was designed to obtain the RMSE of four unknown parameters in the model (viscosity, elasticity modulus, linear velocity, and height correction). In the real data experiment, a stretch of highway in Fuoshan, Guangdong province was selected as the test area. The SBAS algorithm was used to process 16 TerraSAR X high-resolution images. Four unknown parameters of the measured area were estimated, and the time-series deformation results of the measured area were inverted eventually. Through an analysis of the results, we found that the higher the elastic modulus and viscosity were, the lower the deformation was. It can also be concluded that the overall temporal characteristics of the time-series deformation showed a non-linear trend of variation, with a gradual decrease of subsidence velocity in the early stage and a small recovery in the later stage. In order to verify the reliability of the results, the HP-deformation component of the interferogram was analyzed. Compared with the pure linear velocity model, the HP-deformation component of the rheological model was reduced by 34%, indicating that the modeling effect was effectively improved after adding the non-linear rheological component into the model. The external accuracy was evaluated by ground-level measurements, with an RMSE of ±5.0 mm for the proposed model, an improvement of 53% compared with the pure linear velocity model.

In our processing, it was very time consuming to carry out a point-wise genetic search algorithm, so the parameter results for the whole area of Lungui Road and its surroundings were not obtained. Our future study will be focused on the parameter estimation algorithm, in order to improve efficiency and accuracy.

## Figures and Tables

**Table 1:** Comparison of root mean square error (RMSE) for each parameter in the simulated experiment.

Rheological Parameters	E (Mpa)	$\eta \;(10^{6}\;\mathrm{Mpa}\;s.)$	v (mm/y)	∆Z (m)
RMSE	±1.5870 (3.5%)	±0.1741 (0.8%)	±3.5 (1.3%)	±0.29 (5.4%)

**Table 2:** List of the interferometric pairs and their parameters with image number 7 as the master (orbit no. 119, descending).

Image No.	Acquisition Date (yyyy/mm/dd)	Normal Baseline (m)	Temporal Baseline (Days)
1	2014/06/17	−71.50	198
2	2014/08/22	−137.97	132
3	2014/09/13	−286.33	110
4	2014/10/05	−110.85	88
5	2014/10/27	−249.06	66
6	2014/11/18	−74.56	44
7	2015/01/01	0	0
8	2015/02/14	−133.14	44
9	2015/03/08	−106.99	66
10	2015/05/13	−271.51	132
11	2015/06/26	−122.85	176
12	2015/08/09	−149.22	220
13	2015/08/31	−65.63	242
14	2015/09/22	−253.29	264
15	2015/10/14	−159.34	286
16	2015/11/05	−233.83	308
17	2015/11/27	−11.87	330

**Table 3:** RMSE comparison on benchmarks (mm).

	BMK4	BMK5	RMSE
Linear velocity model	±10.3	±11.0	±10.7
Rheological model	±3.4	±6.5	±5.0
